# Decreased detection of ESBL- or pAmpC-producing *Escherichia coli* in broiler breeders imported into Sweden

**DOI:** 10.1186/s13028-020-00532-4

**Published:** 2020-06-22

**Authors:** Oskar Nilsson, Stefan Börjesson, Annica Landén, Christina Greko, Björn Bengtsson

**Affiliations:** 1 Department of Animal Health and Antimicrobial Strategies, National Veterinary Institute (SVA), 751 89 Uppsala, Sweden; 2 Department of Biomedical and Clinical Sciences, Linköping University, Linköping, 58183 Sweden

**Keywords:** Antibiotic resistance, Chickens, Spread

## Abstract

In the spring of 2010, it was discovered that a large proportion of broilers in Sweden were colonized with *Escherichia coli* producing extended-spectrum beta-lactamases (ESBL) or plasmid mediated AmpC (pAmpC). It was hypothesized that the high prevalence was due to transfer from an upper level in the production pyramid and sampling upwards in the production pyramid was initiated. From 2010 to 2019, all shipments (n = 122) of broiler breeders were screened on arrival to Sweden for the occurrence of ESBL- or pAmpC-producing *E. coli* using selective methods. Samples of paper linings from shipments of breeders were cultured on MacConkey agar supplemented with cefotaxime (1 mg/L) after pre-enrichment in either MacConkey broth with cefotaxime (1 mg/L), or from late June 2015 in buffered peptone water without antibiotics. ESBL- or pAmpC-producing *E. coli* was isolated from 43 (35%) of these. Over the years, the proportion of positive imports have decreased and during 2018 and 2019 all imports were negative. In conclusion, the occurrence of ESBL- or pAmpC-producing *E. coli* in broiler breeders on arrival to Sweden has decreased. Such bacteria have not been detected in any shipments since 2017.

## Findings

**Fig. 1:**
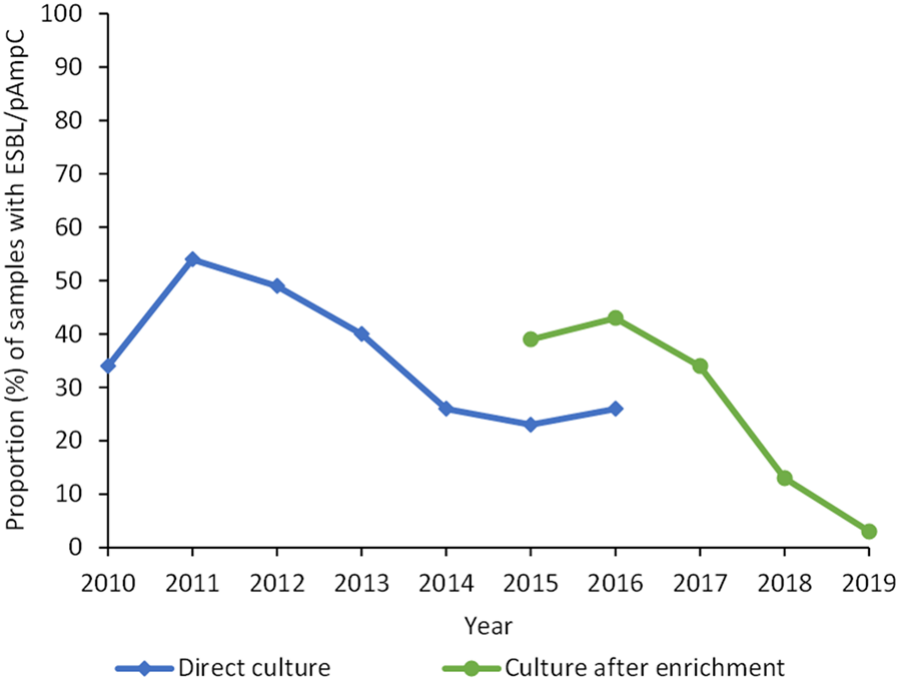
Occurrence of ESBL/pAmpC in broilers in Sweden**.** Proportion (%) of caecal samples from broilers positive for ESBL- or pAmpC-producing *E. coli* from 2010 to 2019. Data from the Svarm program [3]

Enterobacteriaceae producing extended-spectrum beta-lactamases (ESBL) and plasmid-mediated AmpC (pAmpC) are a problem in human clinical settings due to their ability to hydrolyse third generation cephalosporins. There are also indications of potential spread from food producing animals to humans, although only of limited importance in Sweden [1, 2]. Due to the risk of spread from animals to humans via food, the occurrence of *Escherichia coli* with resistance to extended spectrum cephalosporins in caecal samples from broilers has since 2010 been investigated with selective culture methods within the Swedish veterinary antimicrobial resistance monitoring program (Svarm) [3]. In the spring of 2010, it was discovered within the framework of Svarm, that a large proportion of broilers in Sweden was colonized with ESBL- or pAmpC-producing *E. coli* (Fig. 1). Following this finding, the National Veterinary Institute (SVA), in cooperation with the Swedish Poultry Meat Association (SPMA) and the two broiler breeding companies in Sweden started to investigate the sources and reasons for the high prevalence. As the use of antibiotics for broilers in Sweden is low, with less than 1% of raised flocks being treated each year, selection by use of antibiotics was not considered a likely cause [3]. Extended spectrum cephalosporins are not used at all for broilers or broiler breeders in Sweden. Instead, it was hypothesised that the high occurrence was due to transfer from higher level in the production pyramid, as has previously been suggested for other types of antibiotic resistant *E. coli* [4, 5]. Therefore, in the late spring of 2010, sampling upwards in the production pyramid was initiated, starting with environmental samples from the sorting bands in broiler hatcheries and later in hatcheries for parent birds. ESBL- or pAmpC-producing *E. coli* were isolated from both broiler and parent hatcheries indicating introduction via imported day-old breeding stock and subsequent spread vertically and longitudinally in the Swedish broiler production. This hypothesis was reinforced by the findings in a study on vertical transmission conducted from July 2010 to August 2011 [6]. The role of imported breeders for the occurrence of ESBL- or pAmpC producing *E. coli* in national broiler productions has also be demonstrated in other countries [7–10].

As of August 2010, samples of paper linings from all shipments of breeders imported into Sweden by the companies associated to the Swedish Poultry Meat Association (SPMA) have been cultured for ESBL- or pAmpC-producing *E. coli* using selective methods.

According to the protocol suggested by EFSA for monitoring of ESBL- or pAmpC-producing *E. coli* in livestock (www.eurl-ar.eu), adjustments were made in regard to the methodology of the surveillance of imported breeders. Initially all samples were cultured on MacConkey agar with cefotaxime (1 mg/L) after pre-enrichment in MacConkey broth with cefotaxime (1 mg/L), but from late June 2015, the pre-enrichment was changed to buffered peptone water without antibiotics. Suspected cefotaxime resistant *E. coli* were sub-cultured on horse blood agar and verified as *E. coli* by indole test. Detection of genes encoding ESBL- or pAmpC was done using polymerase chain reaction analysis [11–13].

**Fig. 2:**
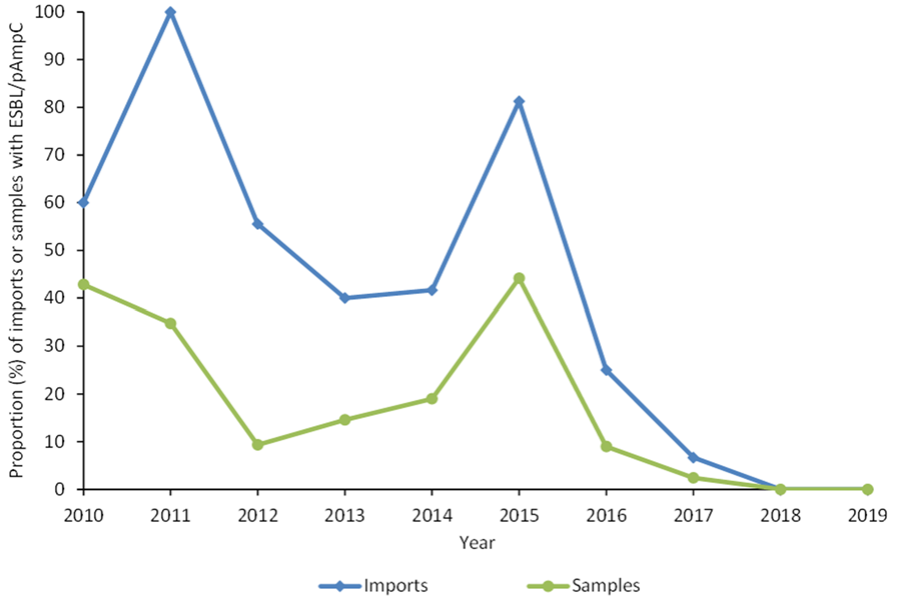
Occurrence of ESBL/pAmpC in broiler breeders imported to Sweden**.** Proportion (%) of shipments and paper lining samples from imported breeders positive for ESBL- or pAmpC-producing *E. coli* from August 2010 to December 2019

From August 2010 to December 2019, 1299 samples of paper linings from breeders originating from 122 shipments have been cultured. At least one sample per breeder line and source farm was sampled, resulting in 4 to 26 samples per shipment. In total, ESBL- or pAmpC-producing *E. coli* was isolated from 195 samples (15%) from 43 (35%) of the shipments. The proportion of samples and shipments positive for ESBL- or pAmpC-producing *E. coli* has varied between the years (Fig. 2 and Table 1), but as of 2017 to 2019 only one shipment has been positive for ESBL- or pAmpC-producing *E. coli*. In general, there has been a decreasing trend since 2010 except for a large increase in positive shipments and samples in 2015. The reason for this temporary increase remains unknown but it was not due to the shift in methodology in June 2015 as the increase was noticed already at the end of 2014, i.e. before the change in methodology. More precisely, between August 2014 to June 2015, 11 out of 14 shipments of breeders where positive for ESBL- or pAmpC-producing *E. coli.* The majority of the isolates carried genes belonging to the *bla*_CMY_-group (n = 146). The remaining isolates carried a gene in the *bla*_CTX-M-1_ -group (n = 36), or *bla*_SHV_ -group (n = 7). Six isolates from two shipments where lost and not available for confirmation. However, the occurrence of pAmpC-producing *E. coli* carrying genes belonging to the *bla*_CMY_-group in the birds from these shipments has been confirmed in subsequent sampling with boot swabs in these flocks (data not shown). Therefore, the original isolates and shipments are considered as ESBL- or pAmpC-positive. Historically, isolates from the Swedish broiler production with a gene in the *bla*_CMY_-group all carried *bla*_CMY-2_, isolates with a gene in the *bla*_CTX-M-1_-group has carried *bla*_CTX-M-1_, and isolates with a gene in the *bla*_SHV_-group has carried the *bla*_SHV-12_ [2, 3, 6].

**Table 1:** Number of shipments positive for ESBL- or pAmpC-producing *E. coli* in total each year per gene groups detected in each shipment

Year	No. of shipments	No. of positive shipments	No. of positive shipments per *bla*-gene group						
			CIT	CTX-M-1	SHV	CTX-M-1 + CIT	CIT + SHV	CTX-M-1 + SHV	ND^a^
2010	5	3	3						
2011	8	8	8						
2012	9	5	3						2
2013	10	4	1	2			1		
2014	12	5	3	1				1	
2015	16	13	9	3		1			
2016	16	4	2	1	1				
2017	15	1	1						
2018	16	0							
2019	15	0							

^a^Not determined: The isolates from these shipments where lost and not available for confirmation. The occurrence of pAmpC-producing *E. coli* carrying genes belonging to the *bla*_CMY_-group in the birds from these shipments has been confirmed in subsequent sampling with boot swabs in these flocks (data not shown)

When assessed on shipment level, the type of genes encoding ESBL- or AmpC have varied over the years (Table 1). However, in the majority of the shipments positive for ESBL- or pAmpC-producing *E. coli* (30/43), all the isolates carried a gene in the *bla*_CMY-2_-gene group.

The exact reasons for the decrease of ESBL- or pAmpC-producing *E. coli* in imported breeding stocks remain unsolved. On the discovery of ESBL- or pAmpC-producing *E. coli* in traded breeders in 2010, discussions between the international companies providing breeders, SPMA and the Swedish companies buying the breeders were initiated by SPMA. Since 2010, the situation regarding ESBL- or pAmpC-producing *E. coli* in the broiler production and potential measures to improve the situation have been discussed regularly between experts from SVA and the Swedish stakeholders, and with the international breeding companies. Possibly, this dialogue that included requests by the Swedish companies that acquired breeders should be free from ESBL/pAmpC-producing *E. coli* and feedback on results have contributed to motivate and encourage the international companies to work towards reducing the occurrence among breeders. The exact measures taken by the international breeding companies are not known to us, except that the previously reported off-label use of cephalosporins at breeder hatcheries has ceased. However, the Swedish breeder companies have requested that cephalosporins should never be used for breeders intended for the Swedish market and hence it is unlikely that any direct selection pressure has been present in those animals.

In conclusion, monitoring of *E. coli* with resistance to extended spectrum cephalosporins in broilers and breeders using selective methods disclosed high occurrence of such bacteria in Swedish broiler production due to transmission from the top of the breeding pyramid. These data underpinned the need to stop transmission by management changes, and that was likely implemented by the broiler industry at the higher levels of the breeding pyramid. Furthermore, the continued monitoring of *E. coli* with resistance to extended spectrum cephalosporins has provided direct feedback on the result of any management changes. The decreased occurrence of ESBL- or pAmpC-producing *E. coli* among breeders is most likely the main reason for the consequent decrease in the occurrence of ESBL- or pAmpC-producing *E. coli* among broilers in Sweden (Fig. 1).

## Data Availability

The datasets used and/or analysed during the current study are available from the corresponding author on reasonable request.

## Abbreviations

ESBL: Extended-spectrum beta-lactamases
EFSA: European Food Safety Authority
pAmpC: Plasmid mediated AmpC
SPMA: Swedish Poultry Meat Association
SVA: National Veterinary Institute, Sweden
